# Genome‐wide analysis of *Pm*TCP4 transcription factor binding sites by ChIP‐Seq during pistil abortion in Japanese apricot

**DOI:** 10.1002/tpg2.20052

**Published:** 2020-08-19

**Authors:** Shahid Iqbal, Zhenpeng Pan, Xinxin Wu, Ting Shi, Xiaopeng Ni, Yang Bai, Jie Gao, Muhammad Khalil‐ur‐Rehman, Zhihong Gao

**Affiliations:** ^1^ Laboratory of Fruit Tree Biotechnology, College of Horticulture Nanjing Agricultural University Nanjing China; ^2^ School of Life Science and Food Engineering Huaiyin Institute of Technology Huai An China; ^3^ Guangdong Provincial Key Laboratory for Plant Epigenetics College of Life Sciences and Oceanography Shenzhen University Shenzhen China

## Abstract

The TCP4 transcription factor plays an important role in plant growth and development, especially in flower development. *Pm*TCP4 is involved in the process of pistil abortion in Japanese apricot, but its molecular mechanism, particularly the DNA binding sites and co‐regulatory genes, are quite unknown. Therefore, to identify the genome‐wide binding sites of *Pm*TCP4 transcription factors and their co‐regulatory genes, chromatin immunoprecipitation sequencing (ChIP‐Seq) was carried out. ChIP‐Seq data produced the maximum enriched peaks in two Japanese apricot cultivars ‘Daqiandi’ (DQD) and ‘Longyan’ (LY), which showed that the majority of DNA‐protein interactions are relevant and have a significant function in binding sites. Moreover, 720 and 251 peak‐associated genes regulated by *Pm*TCP4 were identified in DQD and LY, respectively, and most of them were involved in the flower and pistil development process. Furthermore, Kyoto Encyclopedia of Genes and Genomes (KEGG) analysis showed that photosynthesis and oxidative phosphorylation were the most enriched pathways in both cultivars and all identified genes related to these pathways were down‐regulated. This study will provide a reference for a better understanding of the *Pm*TCP4 regulatory mechanism during pistil abortion in Japanese apricot.

**Abbreviations**
ChIP‐Seq: Chromatin Immunoprecipitation Sequencing
DQD: Daqiandi
IGV: Integrative Genome Viewer
LY: Longyan
MACS: Model‐based Analysis of ChIP‐Seq
MEME: Multiple Em for Motif Elicitation.

## 1 INTRODUCTION

Flower development is a key stage for reproductive success and is one of the most well understood developmental processes in plants (Magallón, Gómez‐Acevedo, Sánchez‐Reyes, & Hernández‐Hernández, 2015). Several diverse mechanisms have been suggested to explain the phenomenon of female sterility and pistil abortion, among which different environmental and genetic factors are generally involved (Beppu & Kataoka, 2011; Zinn, Tunc‐Ozdemir, & Harper, 2010). However, standard environmental conditions, such as ambient temperature, light, and humidity, reduce the problem of pistil abortion to a certain extent (Emms, 1993). *Prunus mume* (Japanese apricot) flowers are classified into two categories: i.e., perfect flowers (normal pistil) and imperfect flowers (pistil absence, pistil browning). Flowers lacking pistils are unable to bear fruit (Gao et al., 2012; Hou et al., 2011). The phenomenon of imperfect flowers is quite common and tremendously affects plant yield; the proportion of these flowers depends on the type of cultivar. Based on our previous studies, the phenomena of pistil abortion in ‘Daqiandi’ and ‘Longyan’ (*P. mume* cultivars) is approximately 76.3% and 5%, respectively (Gao, Wang, & Zhang, 2006). Studies have reported flower abortion and the role of different proteins at different stages of flower development (Shi et al., 2012b). Moreover, numerous metabolic processes, like photosynthesis and production of glucose and starch have also been found to be involved in pistil abortion (Wang, 2008). A morphological study in early‐mid December highlighted that flower buds of Daqiandi stopped pistil differentiation to elongate, leading to abortion (Gao et al., 2012; Shi, Zhang, Gao, Zhang, & Zhuang, 2011). Due to this problem, early morphogenesis of pistil development shows enthusiastic progression comprising a complex series of biochemical and physical changes that further suggest the need for a deep investigation into the pistil abortion mechanism in Japanese apricot (Song et al., 2015). Several morphological studies associated with flower and pistil development are also reported in olive (Reale et al., 2009), *Arabidopsis* (Whittle, Otto, Johnston, & Krochko, 2009), and sweet cherry (Beppu & Kataoka, 2011).

TCP transcription factors are the targets of micro319a (Fahlgren et al., 2007; Sunkar & Zhu, 2004) and have been shown to regulate flower development (Nag, King, & Jack, 2009; Palatnik et al., 2003). Furthermore, they have roles in gametophytic development (Takeda et al., 2006), cell division, cell expansion, the differentiation process during leaf development (Koyama, Furutani, Tasaka, & Ohme‐Takagi, 2007; Nath, Crawford, Carpenter, & Coen, 2003), and auxin biosynthesis (Koyama, Mitsuda, Seki, Shinozaki, & Ohme‐Takagi, 2010), and have previously been reported in peach (Guo et al., 2018b), tomato (Parapunova et al., 2014), and strawberry (Wei et al., 2016). TCP transcription factors encodes a motif, predicted as a basic helix‐loop‐helix (bHLH) structure known as the domain, involved in DNA binding and protein‐protein interaction (Schommer, Debernardi, Bresso, Rodriguez, & Palatnik, 2014). There are two main sub‐classes of the TCP gene family: class I (PCF or TCP‐P) and class II (CYC/TB1 or TCP‐C). Class II is further divided into the CIN sub‐class and CYC/TB1 sub‐class. The main functions of Class I TCP genes, such as PCF1, 2, and 3, are positive regulators of plant cell growth, while class II TCP genes, such as PCF5, 6, 7, 8, CYC, and TB1, are negative regulators of plant cell growth (Martín‐Trillo & Cubas, 2010). In *Arabidopsis*, TCP transcription factor targets are linked with miR319, including TCP2, TCP3, TCP4, TCP10, and TCP24, and thereby these five TCP transcription factors belong to the CIN subclass CIN‐like gene in the CYC/TBI subfamily of class II (Koyama et al., 2007; Palatnik et al., 2003). Several studies have documented that TCP4 transcription factors are the main targets of micro319a and it has been shown that the expression level of the TCP4 gene regulated by micro319a plays a key role in flower development, especially in petal, pistil, and stamen development (Nag et al., 2009; Wang et al., 2017). Consequently, understanding transcriptional regulatory complexes during different plant developmental stages is particularly important and has been focused on for many years. Genome‐wide mapping and DNA‐protein interactions allow us to understand the complete transcriptional regulation network (Johnson, Mortazavi, Myers, & Wold, 2007; Valouev et al., 2008). All biological processes are immediately regulated by a transcription factor (Riechmann et al., 2000; Singh, 1998), thereby, identifying transcription factor binding sites are important for extricating gene regulation during the developmental phases of plants (Shamimuzzaman & Vodkin, 2013).

**Core Ideas**

- Genome‐wide binding sites of *Pm*TCP4 transcription factor during pistil abortion in Japanese apricot studied by ChIP‐Seq
- A significant number of peaks and their associated genes were identified
- This study provides a better understanding of the transcriptional regulatory mechanism during flower and pistil development in Japanese apricot, as well as other related species

Chromatin immunoprecipitation followed by sequencing (ChIP‐Seq) has become a key technique to determine genomic sites of interaction and the corresponding molecular mechanism (Johnson et al., 2007; Reuter, Spacek, & Snyder, 2015). Moreover, ChIP‐Seq offers less noise and larger coverage with high resolution than its array‐based predecessor ChIP‐chip (Laajala et al., 2009; Park, 2009). Due to cost‐effective properties, ChIP‐Seq has become an essential technique to examine gene regulation, transcription factor binding sites, histone modification, and DNA methylation (Aleksic & Russell, 2009; Barski & Zhao, 2009). Previous reports in soybean have depicted a brief description of genome‐wide profiling used for transcription factor binding sites and their regulatory network with the ChIP‐Seq technique (Shamimuzzaman & Vodkin, 2013).

Japanese apricot belongs to the family Rosaceae and is a prominent fruit tree of great importance as an ornamental plant due to its eye‐catching red color (Ni et al., 2018; Zhang et al., 2012). The problem of pistil abortion is very common and affects plant yield in Japanese apricot (Wang et al., 2017), however, the molecular mechanism of this phenomenon is quite unknown. Our previous study in Japanese apricot showed that *Pm‐miR319a* indirectly regulates pistil development by regulating its target gene *Pm*TCP4 (Wang et al., 2017). Therefore, in this study, we investigated the role of *Pm*TCP4 during pistil abortion in Japanese apricot using chromatin immunoprecipitation sequencing (ChIP‐Seq) analysis to gain deep insight into the involvement of downstream genes associated with pistil abortion. Moreover, this study will also help to expand our understanding of the regulatory role of *Pm*TCP4 during flower development in Japanese apricot.

## 2 MATERIALS AND METHODS

### 2.1 Experimental material

The flower buds of Japanese apricot cultivars ‘Daqiandi’ (DQD) and ‘Longyan’ (LY) grown at the National Field Gene‐Bank for Japanese apricot located in Nanjing, Jiangsu Province, China were used as the experimental material. Samples were collected at five different stages (November 14, November 28, December 12, December 26, January 9), with a two‐week interval between stages, as previously described by Wang and Shi (Shi et al., 2011; Wang et al., 2017). The samples were collected in three biological replicates. The collected samples were immediately frozen in liquid nitrogen and then stored at −80°C for further use.

### 2.2 Bioinformatics analysis for molecular characterization of the *Pm*TCP4 protein

The amino acid sequence of *Pm*TCP4 was retrieved from NCBI (https://www.ncbi.nlm.nih.gov/) using the current genome assembly of Japanese apricot (Zhang et al., 2012). The promoter sequence of *Pm*TCP4 was analyzed through the PlantCARE database (http://bioinformatics.psb.ugent.be/webtools/plantcare/html/). The amino acid (aa) number, molecular weight (MW), theoretical isoelectric point, protein hydrophobicity, and the aliphatic amino acid index were determined using the PortParam online tool available from ExPASy (https://expasy.org/proteomics). Subcellular localization was predicted using online software, WoLF PSORT (https://www.genscript.com/wolf-psort.html) and Cell‐PLoc 2.0 (http://www.csbio.sjtu.edu.cn/bioinf/plant-multi/). The protein secondary structure was predicted through SOPMA (https://npsa-prabi.ibcp.fr/cgi-bin/npsa_automat.pl?page=npsa_sopma.html) and protein tertiary structure was predicted using the I‐Tasser online prediction tool (https://zhanglab.ccmb.med.umich.edu/I-TASSER/) and SWISS‐MODEL (https://swissmodel.expasy.org/interactive).

### 2.3 Phylogenetic and Cis‐element analysis

Multiple sequence alignments of the TCP4 protein from Japanese apricot and other plants were performed using muscle alignment and the phylogenetic tree was constructed using the neighbor‐joining method in MEGA 7.0 (Kumar, Stecher, & Tamura, 2016).

The putative *cis*‐elements in the promoter region of the *Pm*TCP4 protein (1500 bp upstream) were annotated using the PlantCARE (http://bioinformatics.psb.ugent.be/webtools/plantcare/html/) database. The elements involved in different processes were summarized.

### 2.4 Detection of the polyclonal antibody against *Pm*TCP4 and western blot analysis

The equilibrium column was washed with PBS (10‐column volume) solution. Subsequently, the antibody‐containing serum was centrifuged at high speed and the supernatant was mixed with an equal volume of 2×PBS buffer; the ion concentration and pH was adjusted. The 10‐column was washed with PBS until no protein was detected in the effluent. Then two‐column volumes of 0.1 M citric acid (citrate acid, adjusted pH 2.7) were added and the flow‐tube was fixed and allowed to stand for 5 minutes to collect the permeate. This was repeated three times. Optical density (280 nm) was used to determine the antibody concentration and purity was determined through SDS‐PAGE. The eluted antibody was added to 2/5 volume of 1 M Tris (pH 8.0) and cut into the desired buffer using the Millipore protein concentrator, normally 2×PBS (containing 0.02% NaN3 and 1 mM EDTA). The purity of the antibody was then checked using SDS‐PAGE and stored at −20°C.

To examine the quality and specificity of the antibody for the ChIP‐Seq assay, a western blot analysis was carried out according to the method of Lee et al. (2016). The samples identical to ChIP‐Seq analysis were used for western blot analysis. Pistils of both cultivars were separated and homogenized and the proteins were extracted in SDS loading buffer. Extracted proteins were separated by running SDS‐PAGE and transferred to a nitrocellulose membrane. After electro‐blotting, proteins were stained with Ponceau solution and blocked with 5% non‐fat milk in 1×PBS for 1 h. Afterward, the membrane was immersed in 5% non‐fat milk with 1×PBS solution containing the primary antibody and was incubated overnight at 4 °C. The membrane was washed and protected with the secondary antibody against HRP‐conjugated goat polyclonal anti‐rabbit IgG (1:1000) for 1.5 h at room temperature. The membrane was washed three times and stained with Enhanced chemiluminescence (ECL) solution. Finally, the results were photographed using the VersaDoc Imaging System (BIO‐RAD).

### 2.5 ChIP‐Seq library preparation and data analysis

Based on previous studies (Shi et al., 2011; Wang et al., 2017), early‐mid December (December 12) was the key time for pistil abortion. Stage 3 (December 12) flower buds were used for ChIP‐Seq analysis. Pistils of both cultivars, DQD and LY, were dissected from flower buds and used for the ChIP‐Seq experiment following the method described by (Kaufmann et al., 2010). In both cultivars, 1.0 g of the pistils were collected and fixated/cross‐linked with 1% formaldehyde in a vacuum for 15 minutes and then homogenized to powdered form in liquid nitrogen. The chromatin was isolated and sonicated to shear the DNA into small fragments (200‐600 bp) using a Digital Probe Sonifier. The sonicated DNA samples were raised with a polyclonal antibody established against the TCP4 transcription factor produced by Synthgene Biotechnology Co., Ltd (China). Samples without the antibody treatment were used as a control. The DNA was purified and ChIP‐Seq libraries were constructed using a KAPA Hyper Prep Kit (KR0961; U.S.A.) following the manufacturer protocol. High‐throughput sequencing using an Illumina HiSeq 3000 Sequencer was performed by Genergy Co., Ltd (Shanghai, China) (Supplemental Figure S1).

The raw reads generated by ChIP‐Seq analysis were then aligned to the reference *P. mume* genome (https://www.ncbi.nlm.nih.gov/genome/?term=prunus%20mume) using the Bowtie aligner (bowtie ‐q  ‐p 48 ‐best ‐m 1 ‐v 2) (Langmead, Trapnell, Pop, & Salzberg, 2009) to match the number of reads to the genome. The experiment was performed as a control (without antibody) and antibody‐treated. MACS (Model‐based Analysis of ChIP‐Seq version 1.4.2) software (Zhang et al., 2008) with standard parameters was used for peak calling to locate the enriched regions of binding sites. Then, the genomic location of the peaks was determined using the *P. mume* database (link provided above) with the Python programming script. The binding peaks were sorted according to the following criteria: (I) if the binding sites exist in the gene body, then it will further be categorized to its location i.e. CDS, intron, exon, 3′ and 5′‐UTR, up2k, down2K, and intergenic region; (II) if the binding site is located in the 1000 bp upstream region and is known as a promoter region; and (III) if neither of these conditions were met then this peak is in an intergenic region. The detected peaks were visualized using the IGV (Integrated Genome Viewer version 2.5.0) genome browser (http://software.broadinstitute.org/software/igv/home). Motif analysis was performed based on the location of detected peaks and enrichment positions using MEME software (Bailey, Williams, Misleh, & Li, 2006). Some of the identified motifs were subjected to JASPAR to be distinguished from other known motifs in the plant transcription factor database.

### 2.6 GO and KEGG pathways enrichment analysis for peak‐associated genes

To analyze the primary biological functions, all peak‐associated genes were mapped to the GO (gene ontology) database (http://www.geneontology.org/) to categorize the genes or their product into different components; i.e., biological process (BP), cellular component (CC), and molecular function (MF) with a fold change ≥ 2 and FDR threshold ≤ 0.001 as a significant enrichment. The KEGG pathway (Kyoto Encyclopedia of Genes and Genomes) (https://www.genome.jp/kegg/pathway.html) with a corrected *p*‐value < .05 was defined as a significantly enriched pathway (Kanehisa et al., 2007). The Blast2GO program (version: v2.5.0) was used to obtained GO annotation and WEGO software (http://wego.genomics.org.cn/) for functional classification. The pathway‐based analysis was carried out to further understand the biological functions of these genes.

### 2.7 RNA extraction and quantitative reverse transcription PCR (RT‐qPCR)

The expression of selected candidate genes was confirmed using RT‐qPCR. Total RNA was extracted from the samples of both cultivars as previously described for the ChIP‐Seq experiment using the Foregene Nucleic Acid Extraction Kit (Foregene Co. Shenzhen, China), following the manufacturer protocol. The first‐strand cDNA was synthesized using Superscript II reverse transcriptase (Invitrogen, San Diego, CA, USA). Gene‐specific primers were designed using the Beacon Designer (version 7.0) software (Premier Biosoft, Palo Alto CA) (Supplemental File S10). RT‐qPCR was performed using the ABI‐7300 Real‐Time PCR system (Applied Biosystems, Foster City, CA, USA) and SYBR Green Master Mix (Toyobo, Osaka, Japan). The experiment was performed using the method described by (Guo et al., 2018a; Iqbal et al., 2020; Wu et al., 2019). Three technical repeats were performed for each biological repeat. The relative expression of the genes was calculated using the 2^−△△CT^ method.

## 3 RESULTS

### 3.1 Molecular characterization of the *Pm*TCP4 protein

The *Pm*TCP4 protein was characterized and had a molecular weight of 47.66 kDa with 439 amino acid (aa) and a theoretical pI of 6.79. Signal peptide and cleavage site prediction of the protein was performed by Signal 4.1. The results showed that the original cleavage site of amino acid residue signal peptide scores were small and did not exceed the 0.5 threshold (Supplemental Figure S2A). Thus, it showed that the *Pm*TCP4 protein does not have a signal peptide and predicted that this may be a non‐secreted protein. The hydrophobicity level of this protein was between −3.089 and 1.722 (Supplemental Figure S2B). It was also predicted that *Pm*TCP4 is a non‐transmembrane protein (Supplemental Figure S2C‐D). Subcellular localization of the *Pm*TCP4 protein was predicted. WoLF PSORT and Cell‐PLoc predicted that this protein is localized in the nucleus.

The secondary structure of the protein acts as a link between the primary and secondary structure of the protein and the prediction of the secondary structure provides more information related to the tertiary structure and function. Here, we used SOPMA online software to predict the *Pm*TCP4 secondary structure. The results showed that the protein secondary structure was comprised of 13.90% ɑ‐helix (Hh), 12.07% extended strand (Ee), 1.59% β‐turn, and 72.44% random coil (Cc). From this analysis, it is speculated that the main structural elements of the *Pm*TCP4 protein are random coils (Supplemental Figure S2E).

Protein tertiary structures are a complex three‐dimensional structure formed through winding and folding of the polypeptide chain based on the secondary structure. The protein tertiary structure formed through the interaction of side‐chain groups by the mean of secondary bonds. Based on the secondary structure of the protein, homology modeling prediction tools (SWISS‐MODEL and I‐Tasser) were used to predict the three‐dimensional structure of the *Pm*TCP4 protein. The SWISS‐MODEL successfully modeled the *Pm*TCP4 protein tertiary structure (Supplemental Figure S2F), while I‐Tasser predicted the highest‐ranking *Pm*TCP4 three‐dimensional structures (Supplemental Figure S2G).

### 3.2 Phylogenetic analysis and *Cis*‐elements in the promoter region of *Pm*TCP4

To determine the evolutionary relationship of *Pm*TCP4 in Japanese apricot and other plants, TCP4 sequences were collected and used to construct the phylogenetic tree using the neighbor‐joining (NJ) method in MEGA 7.0. Phylogenetic analysis showed that *Pm*TCP4 and TCP4 of several other plants belong to the same clades, suggesting that they might have similar biological functions (Supplemental Figure S3).

*Cis*‐elements have an important role in gene transcriptional regulation during different plant developmental processes. Therefore, to understand the transcriptional regulation of *Pm*TCP4, *cis*‐elements in the promoter region of the *Pm*TCP4 were identified. Many common *cis*‐elements, such as CAAT‐box, TATA‐box, and other elements, were identified in the promoter region of *Pm*TCP4 (Supplemental Figure S4). These *cis*‐elements were involved in a variety of processes, such as light‐responsive elements, meristem expression, gibberellin‐responsive elements, anaerobic induction elements, low‐temperature responsiveness, salicylic acid responsiveness, auxin‐responsive elements, and MeJA responsiveness. Therefore, it might be speculated that *Pm*TCP4 is involved in a variety of environmental and developmental changes and processes that shows its specific role in plant development.

### 3.3 ChIP‐Seq libraries and peaks detection

Prior to ChIP‐Sequencing, the quality and specificity of the antibody was checked. Pistils of DQD and LY were used to validate the expression or specificity of the *Pm*TCP4 protein through western blot analysis (Supplemental Figure S5). ChIP‐Seq libraries were constructed from the pistils of *P. mume* cultivars DQD and LY. After crosslinking the samples, chromatin was isolated and sonicated to a suitable fragment size for sequencing analysis. Afterward, the ChIP‐Seq libraries were constructed and high‐throughput sequencing was performed. In DQD ChIP‐Seq libraries, a total of 41.5 million raw reads were obtained for antibody‐treated samples and 51.2 million raw reads for the control samples of three biological replicates. Similarly, in LY ChIP‐Seq libraries, we obtained a total of 41.3 million raw reads for antibody‐treated samples and 42.0 million raw reads for the control samples of three biological replicates (Table 1; Supplemental File S1).

**TABLE 1:** Summary of ChIP‐Seq reads from twelve different libraries matched to the *Prunus mume* genome

Name of cultivar	Sample type	Raw reads	Clean reads	Genome mapped reads
Daqiandi	Control	51287674	49971091	42034200
	Anti‐body treated	41501250	39437936	15325300
Longyan	Control	42036358	40915584	35257642
	Anti‐body treated	41328049	29205209	6355283

**FIGURE 1:**
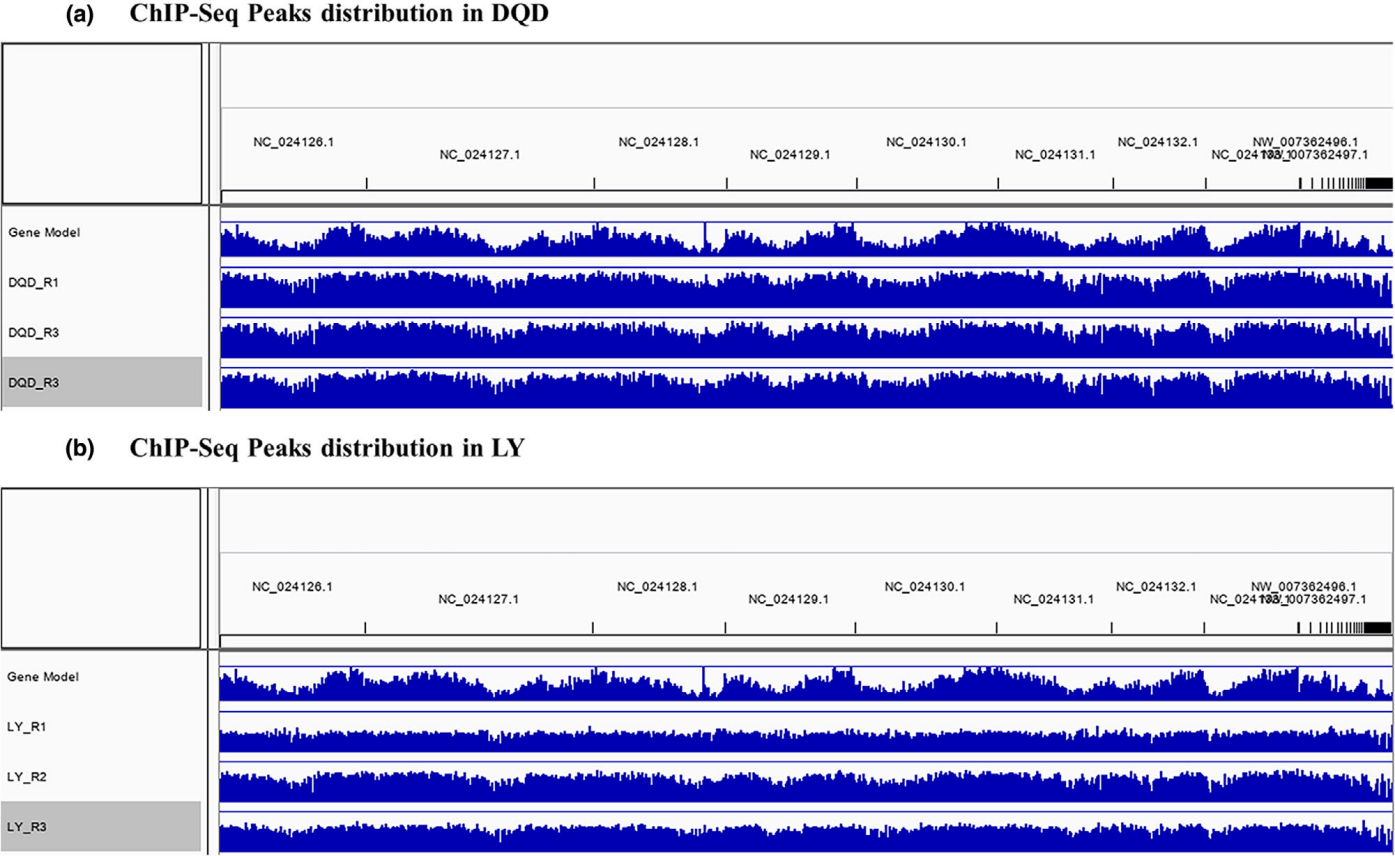
An overview of ChIP‐Seq peak distribution in the *Prunus mume* genome chromosomes. (a) Distribution of ChIP‐Seq peaks throughout *P. mume* chromosomes in cultivar Daqiandi (b) Distribution of ChIP‐Seq peaks throughout *P. mume* chromosomes in cultivar Longyan

Raw reads obtained from ChIP‐Seq were aligned to the *P. mume* reference genome using Bowtie software (bowtie ‐q ‐p 48 ‐best ‐m 1 ‐v 2) to match the reads to the genome. Several peak detecting procedures were used on ChIP‐Seq data sets. MACS was used for peak calling analysis to identify the enrichment binding sites. MACS identified 1049 enriched unique peaks in DQD (Supplemental File S2) and 471 enriched unique peaks in LY (Supplemental File S3) with a *p‐value* < .05. The distribution of detected peaks for *Pm*TCP4 in both cultivars was visualized using IGV software (Figure 1).

### 3.4 Genomic location of ChIP‐Seq peaks and their associated genes regulated by *Pm*TCP4 transcription factor

**FIGURE 2:**
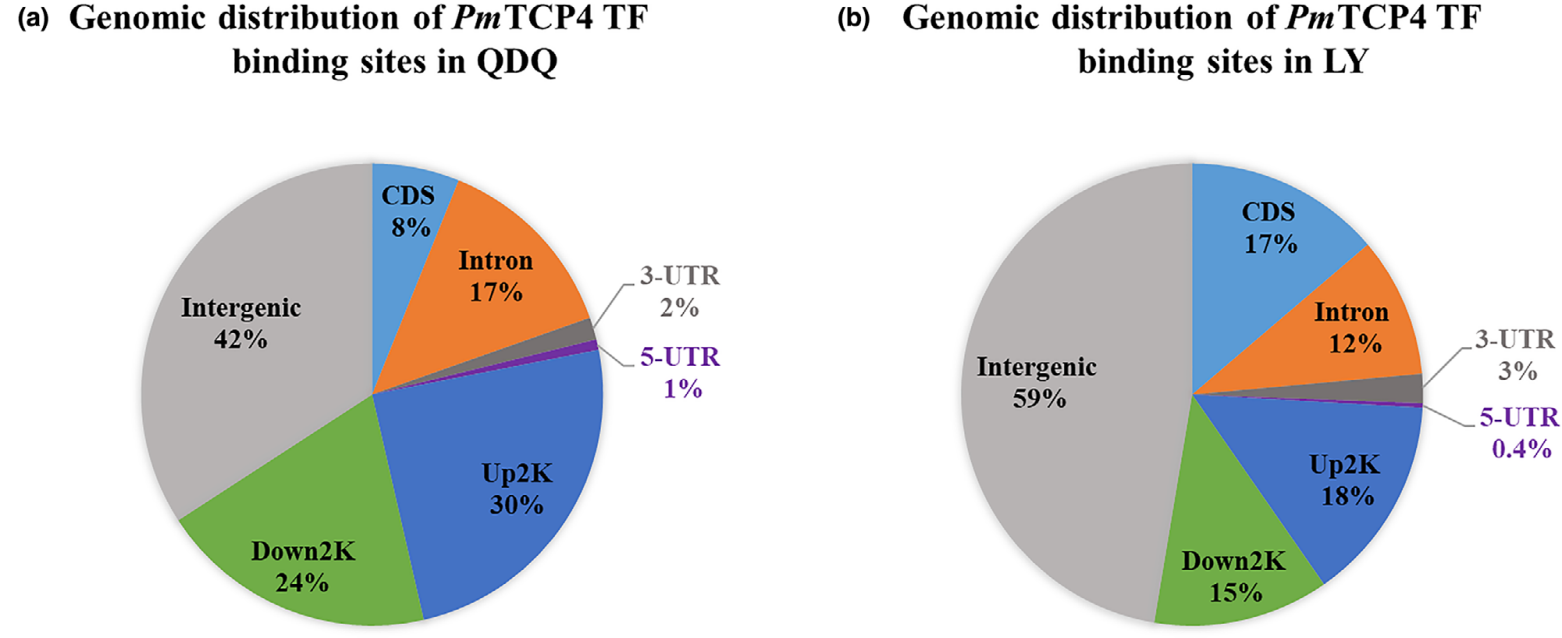
An overview of the genomic distribution of *Pm*TCP4 transcription factor binding sites. (a) The pie chart shows the genomic distribution of ChIP‐Seq peaks for the *Pm*TCP4 transcription factor binding sites associated with its genome in cultivar Daqiandi (b) The pie chart shows the genomic distribution of ChIP‐Seq peaks for the *Pm*TCP4 transcription factor binding sites associated with its genome in cultivar Longyan

**FIGURE 3:**
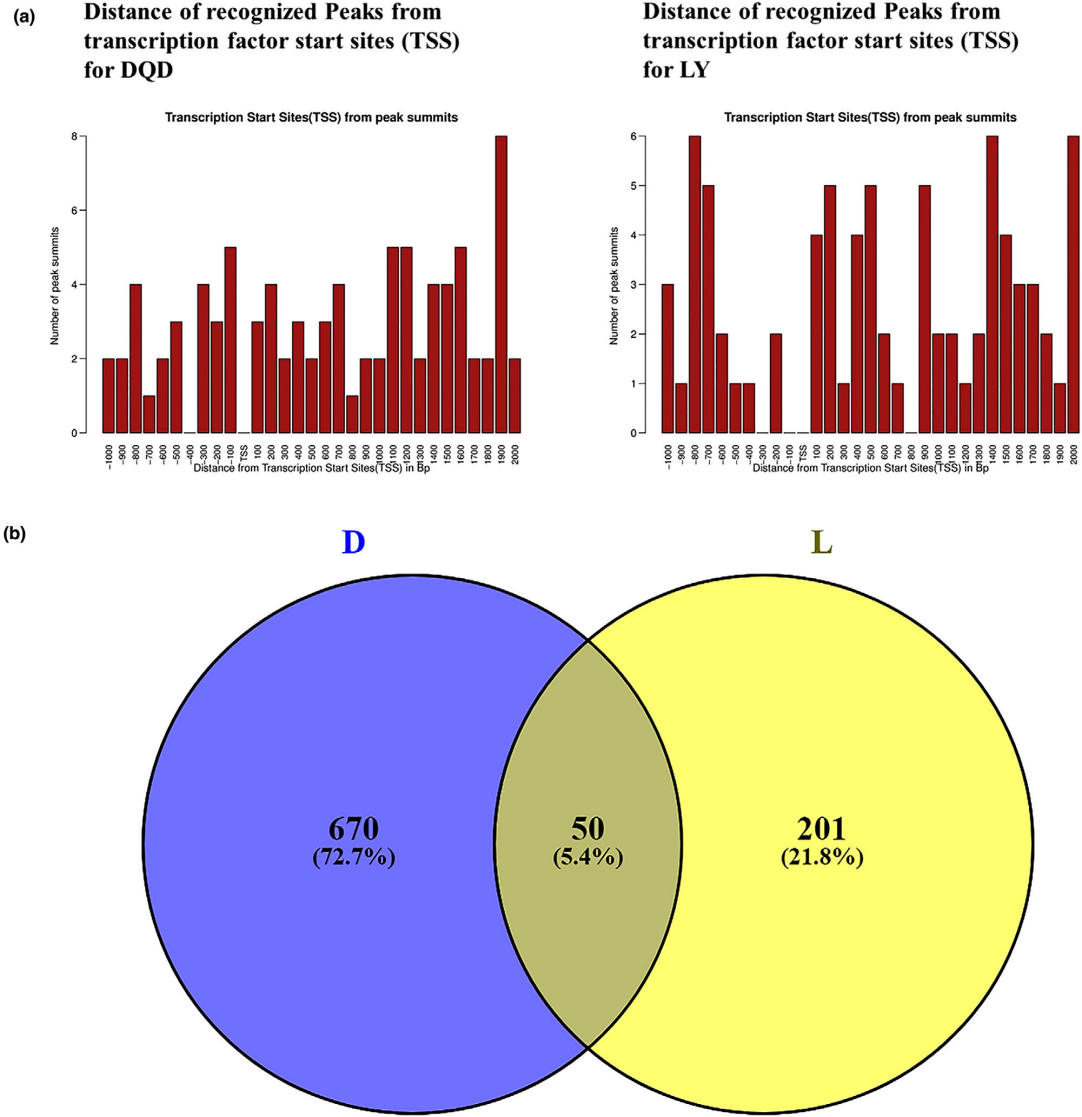
Highly enriched regions of *Pm*TCP4 transcription factor binding sites and their peak‐associated genes. (a) The distance between transcription factor start sites (TSS) and MACS peak summits for genome associated peaks. This figure shows the peaks found up to 1000 bp upstream and 2000 bp downstream of TSS in Daqiandi (DQD) and Longyan (LY) and are highly enriched in the promoter region (b) Venn‐diagram of peak‐associated genes in DQD and LY cultivars of Japanese apricot

The genomic location of the detected peaks of DQD and LY were identified using a custom Python (v 3.7.4) programming script from the *P. mume* genome annotation. We found that a significant number of peaks were located in the promoter region. In DQD, peaks were located as in the intergenic region (42%), Up2K/Promoter region (30%), Down2K (24%), intron (17%), CDS (8%), 3‐UTR (2%), and 5‐UTR (1%). Similarly, in LY, peaks were located in the intergenic region (59%), Up2K/Promoter region (18%), Down2K (15%), intron (12%), CDS (17%), 3‐UTR (3%) and 5‐UTR (0.4%) (Figure 2). Furthermore, these peaks were also found to be located adjacent to the transcription start site (Figure 3a). Moreover, ChIP‐Seq data identified 720 and 251 potential peak‐associated genes in DQD and LY, respectively (Supplemental Files S4 and S5), that were regulated by the *Pm*TCP4 transcription factor (Figure 3b).

### 3.5 Motif analysis

Motif analysis was performed using MEME software. For MEME analysis, the most enriched peaks of both cultivars were used. The motif analysis discovered five new motifs for DQD, in which motif‐3 matched with the *SOC1* motif in the plant transcription factor binding motifs database of JASPAR CORE plants (Sandelin, Alkema, Engström, Wasserman, & Lenhard, 2004). Similarly, in LY, 5 novel motifs were discovered but no one was matched to the JASPAR CORE database (Supplemental Figure S6).

### 3.6 GO and KEGG functional enrichment analysis for peak‐associated genes

**FIGURE 4:**
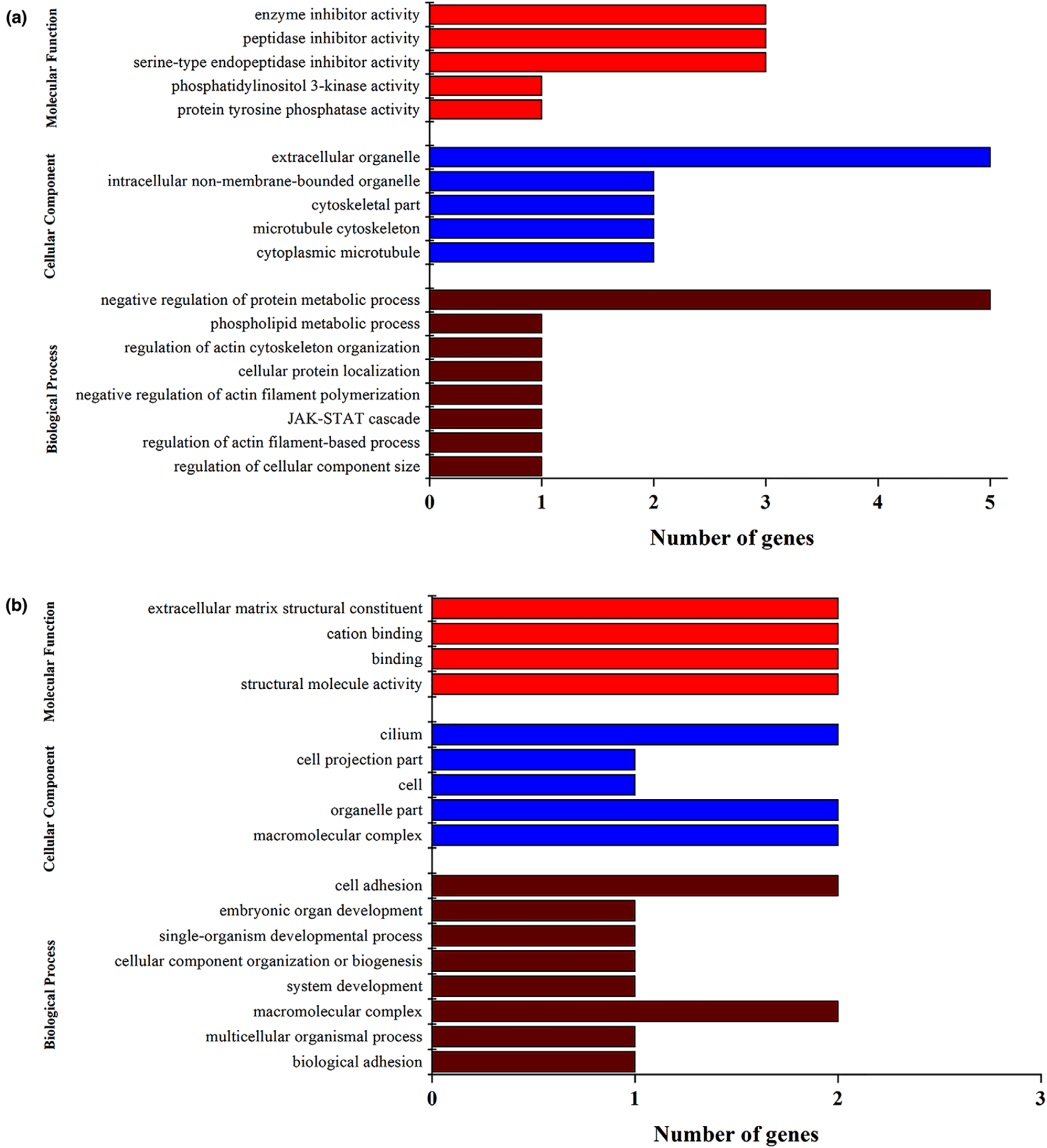
Gene ontology distribution of peak‐associated genes. (a) shows the GO‐terms of cultivar Daqiandi (b) shows the GO‐terms of cultivar Longyan

To understand the biological functions of downstream genes during pistil abortion in the Japanese apricot, GO functional enrichment analysis was performed, using a *p*‐value ≤ .05 as a threshold. In DQD, the GO enrichment analysis identified 209 enriched functional classes for biological process, which primarily were: regulation of cellular component size, regulation of actin filament‐based process, and JAK‐STAT cascade. The cellular component recognized 54 enriched functional classes and were primarily: cytoplasmic microtubule, cytoskeletal part, and intracellular non‐membrane‐bounded organelle. The molecular function covered 27 enriched classes and associated enrichment terms were: protein tyrosine phosphatase activity, phosphatidylinositol 3‐kinase activity, and enzyme inhibitor activity (Figure 4a; Supplemental File S6). In LY, the GO enrichment analysis identified 66 enriched functional classes for biological process through which most enriched terms were: cellular component organization or biogenesis, single‐organism developmental process, and embryonic organ development. The cellular component recognized 36 enriched functional classes and primarily were: macromolecular complex, cell, and cilium. The molecular function covered 6 enriched classes and associated enrichment terms were: structural molecule activity, binding, and extracellular matrix structural constituent (Figure 4b; Supplemental File S7).

**FIGURE 5:**
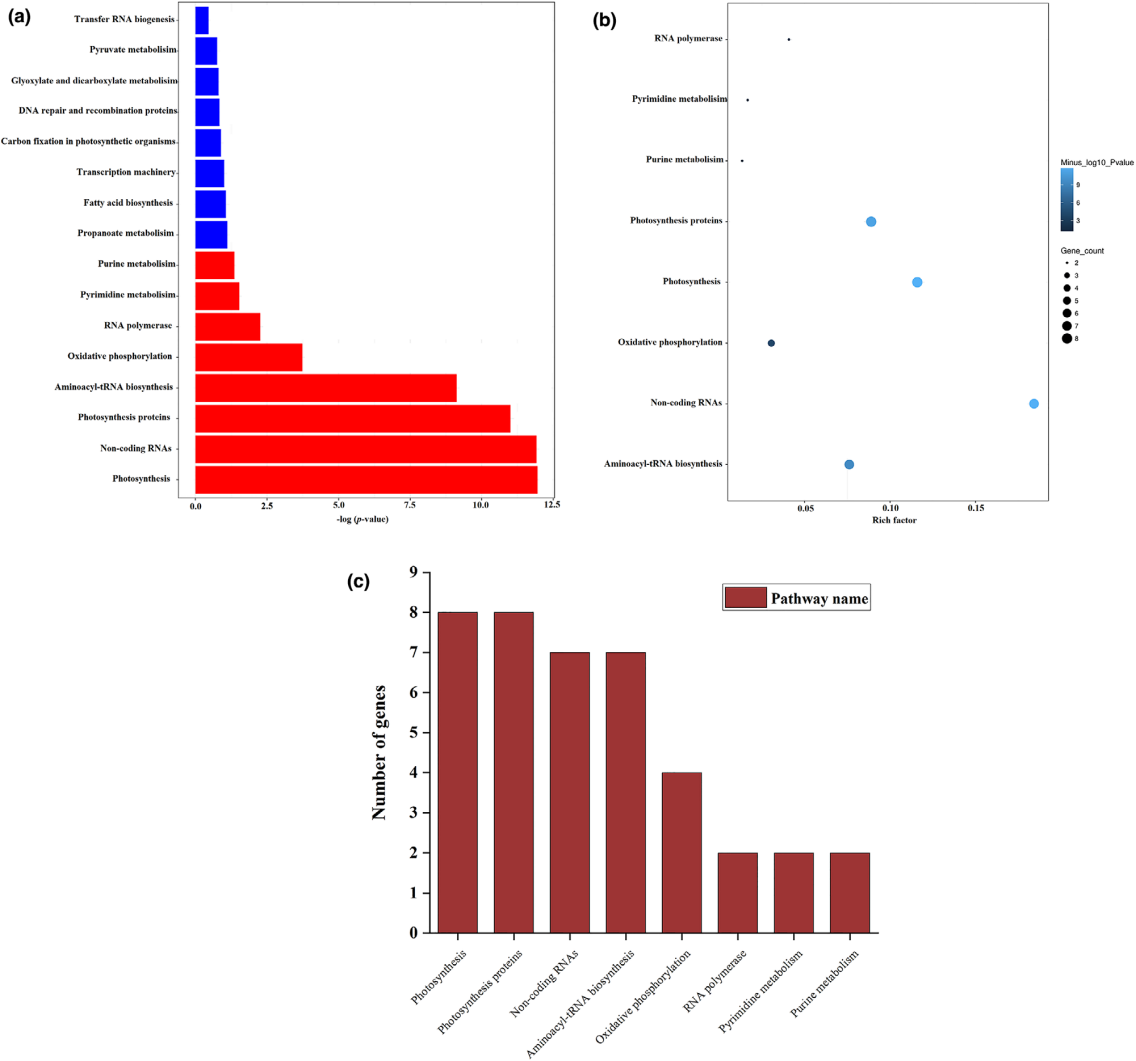
Common significantly enriched pathways. (a) A histogram shows a significant enriched pathway‐level based on –log(*p*‐value) (b) The bubble chart represents the pathway significance level. The size of the bubble shows the significance level (c) The number of genes in the significantly enriched pathway

In KEGG pathway analysis, a total of 20 enriched pathways were identified in DQD (Supplemental File S8) and 7 enriched pathways were identified in LY (Supplemental File S9). The five significant enriched pathways were photosynthesis, photosynthesis proteins, oxidative phosphorylation, pyruvate metabolism, and fatty acid biosynthesis and were common in both cultivars (Figure 5). The genes related to these pathways have (or are near) binding sites for *Pm*TCP4.

In the present study, the results revealed that three pathways, including photosynthesis (pmum00195), photosynthesis protein (pmum00194), and oxidative phosphorylation (pmum00190), were significantly enriched during pistil abortion in both cultivars. All identified genes associated with these pathways were downregulated (Table 2).

**TABLE 2:** Common enriched pathways for peak‐associated genes in cultivars Daqiandi and Longyan

Pathway ID	Pathway name	Differently expressed gene symbols	FDR
Daqiandi			
pmum00195	Photosynthesis	atpA, atpF, atpH, atpI, LOC103344104, petA, petB, petD, petN, psaI, psbA, psbI, psbK, psbM, atpB, LOC103342490	7.07×10^−16^
pmum00194	Photosynthesis proteins	atpA, atpF, atpH, atpI, LOC103344104, petA, petB, petD, petN, psaI, psbA, psbI, psbK, psbM, atpB, LOC103342490	3.17×10^−14^
pmum00190	Oxidative phosphorylation	atpA, atpF, atpH, atpI, LOC103344104, ndhF, atpB, LOC103335303, LOC103342942, LOC107881883, LOC107881951, LOC103331497, ndhC, ndhJ, ndhK, LOC103320535, LOC103323534, LOC103327611	7.14×10^−3^
pmum00620	Pyruvate metabolism	LOC103321348, LOC103337187, LOC103325935, LOC103330440, LOC103335532, LOC103339581, accD, LOC103326102, LOC103328478	2.25×10^−1^
pmum00061	Fatty acid biosynthesis	accD, LOC103322589	9.54×10^−1^
Longyan			
pmum00195	Photosynthesis	atpA, atpF, petN, psbA, psbM, atpH, atpI, LOC103344104, LOC107880548, psaI, psbI, psbK, atpB, psbB, psbD, psbH, psbT, petB, petD	7.07×10^−16^
pmum00194	Photosynthesis proteins	atpA, atpF, petN, psbA, psbM, atpH, atpI, LOC103344104, psaI, psbI, psbK, atpB, psbB, psbD, psbH, psbT, petB, petD	3.17×10^−14^
pmum00190	Oxidative phosphorylation	atpA, atpF, atpH, atpI, LOC103344104, ndhF, LOC103335651	7.14×10^−3^
pmum00620	Pyruvate metabolism	accD	2.25×10^−1^
pmum00061	Fatty acid biosynthesis	accD, LOC103322589	9.54×10^−1^

### 3.7 Dynamic expression of genes identified by ChIP‐Seq

To analyze the gene regulatory network of a transcription factor, it is necessary to study the expression pattern of the genes. In this study, twelve genes related to photosynthesis and oxidative phosphorylation pathways were selected to observe the expression level in two cultivars. RT‐qPCR results revealed highly downregulated expression in DQD when compared to LY, as determined by ChIP‐Seq analysis (Supplemental Figure S7).

## 4 DISCUSSION

Japanese apricot (*Prunus mume* Seib. et. Zucc) is an important fruit and ornamental plant with great economic importance as a preserved or value‐added product (Ni et al., 2018). Flower development is an important stage of plant growth and development and is affected by various environmental and genetic factors. In the flower, pistil development plays a substantial role during fruit formation and development, which ultimately contributes toward plant yield. Many flower responsive genes and transcripts have been identified in previous studies (Wollmann, Mica, Todesco, Long, & Weigel, 2010). Recently, ENCODE (Encyclopedia of DNA Elements) used different human cell lines to generate ChIP‐Seq data sets for a large number of transcription factors to identify genome‐wide functional and regulatory DNA elements throughout the genome (Consortium, 2012; Gerstein et al., 2012). In *Arabidopsis*, a large number of transcription factor binding sites have been identified using ChIP‐Seq (Immink et al., 2012; Oh et al., 2009). Several miRNAs have been reported to play a substantial role in regulating floral organ development (Luo, Guo, & Li, 2013). In *Arabidopsis*, TCP4, a target of miR319a, is critical for petal growth and anther development (Nag et al., 2009). Our previous study demonstrated the role of TCP4 during pistil abortion in Japanese apricot (Wang et al., 2017). Therefore, in this study, to identify *Pm*TCP4 binding sites and downstream genes in Japanese apricot during pistil abortion, a combination of experimental and bioinformatics approaches was used. To our knowledge, this is the first‐ever report to study the role of *Pm*TCP4 binding sites during pistil abortion in Japanese apricot using the ChIP‐Seq technique.

In this study, the pistils of *P. mume* cultivars DQD and LY were used for a ChIP‐Seq experiment with a specific antibody. The analysis of ChIP‐Seq libraries identified 1049 and 471 highly enriched peaks for DQD and LY, respectively, using MACS software at a significance level of *p *< .05. A significant number of these peaks were associated with the *P. mume* gene model. We found that in both cultivars, a significant number of the peaks were located in the promoter region of the *P. mume* gene model and are consistent with a previous study (Shamimuzzaman & Vodkin, 2013). These results suggested that the majority of DNA‐protein interactions identified in the ChIP‐Seq experiment are relevant and the function of these binding sites have significant importance in the enrichment of GO annotations among *Pm*TCP4 targets. In MEME analysis, the highly enriched peaks of promoter regions were selected in DQD, and five common DNA motifs were found, from which one of the motifs matched to *SOC1*. In LY, we also found five common DNA motifs, but no motifs matched other species.

In DQD and LY, the GO terms found in different developmental processes that are largely involved in plant growth and organ development were previously reported in flower and organ development in plants (Habu & Tao, 2013; Shearman et al., 2013). In our study, the KEGG analysis identified 20 enriched pathways for DQD and 7 enriched pathways for LY. In both cultivars, five pathways, including photosynthesis, photosynthesis proteins, oxidative phosphorylation, pyruvate metabolism, and fatty acid biosynthesis, were commonly enriched, from which the photosynthesis and oxidative phosphorylation pathway are vital and closely related to flower and pistil development. Photosynthesis is an important source for plant organ development, especially during flower development. Improper photosynthesis processes cause the plant to inhibit proper organ elongation by limiting cell enlargement and cell division. In flowers, improper photosynthesis (very low or very high light intensity) causes fertility problems (Dell & Huang, 1997). Furthermore, a sufficient amount of photosynthesis is necessary for generative development of the flower (Kandeler, Hügel, & Rottenburg, 1975). In the photosynthesis pathway, the identified genes were mostly associated with ATP synthase (atpA, atpB, atpF, atpH, and atpl), cytochrome (petA, petB, petD, and petN), and the photosystem (psbA, psbB, psbD, psbH, psbK, psbl, psbM, psbT, and psal) and have been previously discussed in *Arabidopsis* reproductive development (Sotelo‐Silveira et al., 2013). In addition, transcripts encoding ATP synthase (atpA, atpB, atpH, atpH, and atpl) and NADH‐ubiquinone oxidoreductase (ndhC, ndhF, ndhJ, and ndhK) in the oxidative phosphorylation pathway were found to regulate reproductive organ development (Shi et al., 2012a). Similar findings related to floral development and pistil abortion have already been reported in *Cymbidium sinense* (Zhang et al., 2013), *Litchi chinensis* (Liu, Wang, Chen, & Zhou, 2019), and chickpea (Singh, Garg, & Jain, 2013). However, the genes showed highly downregulated expression in DQD compared to LY, suggesting that these genes might play an important role in pistil abortion (Supplemental Figure S8). Our ChIP‐Seq results indicate that *Pm*TCP4 transcription factor binding sites and their downregulated genes have a great influence during flower and pistil development in Japanese apricot.

## 5 CONCLUSION

In the present study, we comprehensively investigated the role of *Pm*TCP4 during pistil abortion using ChIP‐Seq analysis in Japanese apricot. Our results related to the *Pm*TCP4 transcription factor binding sites represent a better understanding of the regulatory mechanism during pistil abortion. The identification of DNA motifs and regulatory genes provide a new approach to understanding the molecular mechanism of *Pm*TCP4 during pistil abortion. Overall, these results improve our understanding of the genetic mechanism underlying transcriptional regulation and functional characterization of candidate downstream genes involved in the flower development process.

## AUTHOR CONTRIBUTIONS

ZG and SI conceived and designed the study. SI and PP performed the experiment. SI analyzed the data and wrote the manuscript. WX, NX, YB, and JG participated in sample collection and data analysis. TS and M.K.R revised the manuscript. All authors read and approved the final version of the manuscript.

## CONFLICT OF INTEREST DISCLOSURE

The authors declare that they have no conflict of interest.

## Supporting information


**Figure S1**. Complete flowchart for ChIP‐Seq analysis in Japanese apricot (A) Different steps of the ChIP‐Seq experiment for sample preparation (B) Standard workflow for ChIP‐Seq data analysis

**Figure S2**. Molecular characterization and bioinformatics analysis of the *Pm*TCP4 protein in Japanese apricot

**Figure S3**. Phylogenetic analysis of *Pm*TCP4 from Japanese apricot and other Rosaceae plants

**Fig S4**. Type and number of *cis*‐promoter elements of *Pm*TCP4 involved in different processes.

**Figure S5**. Western blot analysis for protein identification in samples

**Figure S6**. Motif analysis of *Pm*TCP4 using the MEME online tool. (A) Novel significant motifs for DQD (B) Novel significant motifs for LY

**Figure S7**. Relative expression level of selected genes by RT‐qPCR

**Figure S8**. Peak length distribution and peak depth distribution of Daqiandi and Longyan

Table S1. Statistics for ChIP‐Seq libraries of two Japanese apricot cultivars 'DQD' and 'LY'

## Data Availability

All data related to ChIP‐Seq have been deposited to NCBI‐GEO under the accession number “GSE132994”.
